# Effects of Colchicine on *Populus canescens* Ectexine Structure and 2n Pollen Production

**DOI:** 10.3389/fpls.2020.00295

**Published:** 2020-03-17

**Authors:** Qing Zhou, Jian Wu, Yaru Sang, Zhengyang Zhao, Pingdong Zhang, Meiqin Liu

**Affiliations:** ^1^Beijing Advanced Innovation Center for Tree Breeding by Molecular Design, Beijing Forestry University, Beijing, China; ^2^National Engineering Laboratory for Tree Breeding, Beijing Forestry University, Beijing, China; ^3^Key Laboratory of Genetics and Breeding in Forest Trees and Ornamental Plants, Ministry of Education, Beijing Forestry University, Beijing, China; ^4^School of Bioscience and Biotechnology, Beijing Forestry University, Beijing, China

**Keywords:** *Populus canescens*, colchicine, chromosome doubling, 2n pollen viability, triploid

## Abstract

Triploid breeding is a central way to improve growth traits, timber quality, and stress resistance in *Populus*. In the present study, the morphology and viability of colchicine-induced 2n pollen, triploid production by crossing induced 2n pollen, and identification of genetic constitution of colchicine-induced 2n pollen were conducted in *Populus canescens* based on optimizing technology for inducing chromosome doubling in pollen. We found that the meiotic stage, injection time, and the interaction between the meiotic stage and injection time had highly significant effects on the 2n pollen production rate. The most effective treatment for inducing 2n pollen was to give 11 injections of 0.5% colchicine solution when pollen mother cells (PMCs) were at the pachytene stage. The highest 2n pollen production rate was 30.27 ± 8.69%. Colchicine occasionally affected ectexine deposition, and some narrow furrows were detected in the ectexine structure. However, no significant difference was observed in the pollen germination rate between natural 2n pollen and colchicine-induced 2n pollen. Moreover, 5 triploids derived from FDR-type 2n pollen were generated by crossing induced 2n pollen, suggesting that colchicine does not eliminate the function of colchicine-induced 2n pollen. However, slower growth of 2n pollen tubes was responsible for a lower triploid production rate.

## Introduction

*Populus canescens* (section *Populus*, family Salicaceae, genus *Populus*) grows in the Irtysh River Basin, Xinjiang Uygur Autonomous Region, in northwest China. Due to its high ornamental value, resistance to disease, insects, and drought, it is widely used for ecological protection in northwest China (Xu, 2008). However, the annual growth rate of *P. canescens* is lower owing to its shorter growing season. A long-term breeding program was developed by Beijing Forestry University to improve *P. canescens* growth. In 1989, a cross-breed between *Populus tomentosa* × *Populus bolleana* and *P. canescens* was first reported by Li and Zhu (1989), and six superior hybrid clones were developed. In a subsequent study, Duan and Yang (1998) evaluated the cold and drought resistance of these superior hybrids by the anatomical structure of the leaves, water loss rate through the leaves, and electroconductivity of the shoots. Tian J. et al., 2018 systematically studied the abnormal behavior of chromosomes during meiosis of pollen mother cells (PMCs), pollen morphology and pollen abortion in some male hybrids between *P. tomentosa* × *P. bolleana* and *P. canescens*.

Triploid breeding is one of most powerful approaches to improve the genus *Populus*. The first discovered triploid (2n = 3x = 57) individual *Populus* was a giant form of *Populus tremula* from a natural population in Sweden, which was characterized by extremely large leaves and rapid growth (Nilsson-Ehle, 1936; Müntzing, 1936). Zhu et al. (1998) reported that genetic gains in the stem volume of 9-year-old natural *P. tomentosa* triploid clones was higher (56.4%) than that of the diploid counterparts. In another study, Zhu et al. (1995) documented that the stem volume of the allotriploid clone B301 was 3.5 times higher that of *P. tomentosa* diploids. Subsequently, Zhang et al. (2014a) reported that the fiber length of triploid white poplar hybrid clones was 20.6% larger than that of diploids. The holocellulose of triploid white poplar hybrid clones was higher 1.3% than that of its diploid counterparts. However, the lignin content of triploid white poplar hybrid clones was shown to be 21.7% lower than that of diploid individuals (Zhang et al., 2014b). Therefore, triploid breeding has not only improved the growth rate but also the wood quality of *Populus* (Zhang et al., 2012, 2013, 2015a,b).

Few studies have focused on *P. canescens* triploid breeding. Only Tian M. et al. (2018) reported 2n pollen production induced by high-temperature exposure and 42 triploids were obtained. Subsequently, the 2n pollen *P. canescens* high-temperature-induced was demonstrated to have originated from second division restitution (SDR) during the meiosis of PMCs. The lower heterozygosity transmitted from the male parent provides SDR-type 2n gametes a lower breeding value than first division restitution (FDR)-type 2n gametes (Veilleux, 1985; Dong et al., 2015). Thus, crossing FDR-type 2n gametes with haploid gametes to create *P. canescens* triploids is a promising way to increase growth rate.

The objective of this study was to produce FDR-type 2n pollen by colchicine induction and generate a triploid *P. canescens* germplasm. To optimize the pollen chromosome doubling technology to produce 2n pollen using colchicine, pollen morphology, 2n pollen germination *in vitro*, and genetic constitution identification were conducted, followed by triploid production using colchicine-induced 2n pollen. Our findings will lay the foundation for the triploid breeding of *P. canescens*.

## Materials and Methods

### Plant Materials

*Populus canescens* and *Populus hopeiensis* are native tree species in northwestern China. *Populus alba* × *Populus glandulosa*, a hybrid with good fertility under normal conditions, was introduced from Korea in 1984. Male floral branches of *P. canescens* “YHY1” were collected from a natural population in Aletai, Xinjiang Uygur Autonomous Region. Female branches of *P. hopeiensis* “HBY1” were collected from the Baotou, Inner Mongolia Autonomous Region, and female branches of *P. alba* × *P. glandulosa* “YXY1” were collected from the Guan Country Nursery, Shandong Province. All sampled floral branches were trimmed and cultured in tap water at the Beijing Forestry University greenhouse (10–20°C). No additional nutrition was added.

### 2n Pollen Induction With 0.5% Colchicine Solution

Every 2 h, 2–3 flower buds were randomly collected and fixed in Carnoy’s solution (ethanol: acetic acid, 3:1). The anthers were dissected from the fixed buds using forceps and were crushed in a droplet of aceto-carmine solution (2%) onto a microscope slide to observe meiosis. When the dominant meiotic stage of the PMCs were the leptotene, pachytene, diplotene, diakinesis, and metaphase I stages. The male buds were injected with 0.5% colchicine solution 3, 5, 7, 9, and 11 times, respectively. The interval between neighboring injections was 2 h. Each bud was injected with 10 μl colchicine solution, and 15–20 flower buds with each treatment were injected. Untreated male buds were taken as a control group. The treated and the control floral branches were hydroponically cultured in a greenhouse for 4 weeks until the catkins matured, then pollen samples were collected and stored in a centrifuge tube with allochronic silica gel at −20°C.

The diameter of the 2n pollen grain is larger than that of haploid pollen grain because of its chromosome doubling in higher plants (De Storme et al., 2012; Zhang and Kang, 2013; Yang et al., 2015). Therefore, we can determine 2n pollen from haploid pollen by the diameter of pollen grains. Thus, the frequency of 2n pollen was estimated according to the method described by Zhang and Kang (2013). The diameters of 200–300 pollen grains per sample were measured.

### Morphology of Colchicine-Induced 2n Pollen Grains

For the scanning electron microscopic analysis, pollen samples were sputter-coated with gold for 1 min, using a Hitachi E-010 ion sputter coater (Tokyo, Japan). The pollen grains were observed under a Hitachi S-3400N microscope at an accelerating voltage of 5 kV. The details of ectexine structure and deposition were observed and photographed for each pollen sample.

### Pollen Germination *in vitro*

The *in vitro* pollen germination medium was prepared according to the method described by Zhao et al. (2017). The medium was composed of 0.7% agar, 50 mg l^–1^ calcium chloride, and 120 mg l ^–1^ boric acid. The pH value of the medium was 6.0. Some fresh colchicine-induced 2n pollen grains were sampled and spread on slides containing the medium. Then, the slides were placed in 120 mm Petri dishes with wet filter paper, and the pollen was germinated in a climate chamber at 26°C. After a 4 h culture, the pollen grains were washed in a liquid germination medium, collected in a 1.5 ml centrifuge tube, and fixed in Carnoy’s solution for 10 min after being centrifuged for 5 min at 1,000 rpm. The germination rate and the length of each sample were calculated using an eyepiece micrometer. A total of 20–30 pollen tubes was assessed in each sample.

### Triploid Production by Crossing Colchicine-Induced 2n Pollen

The female flower buds were pollinated with fresh colchicine-induced *P. canescens* pollen when the stigmas of the female flower buds were receptive. After pollination, the female flower branches were hydroponically cultured. Seeds were collected after 4 weeks and sown into 54 × 28 × 10 cm nutrition plates at a depth of 5 cm to promote growth, then placed in a greenhouse.

### Ploidy Analysis via Flow Cytometry and Somatic Chromosome Counting

A flow cytometry analysis was conducted by flow cytometer (BD FACSCalibur; Becton Dickinson, Brea, CA, United States). Approximately 0.5 g of chopped young leaves were put into a 55 mm Petri dish with 1 ml of nuclear extraction solution (45 mM MgCl_2_, 30 mM sodium citrate, 20 mM 4-morpholinepropane sulfonate, 1% (v/v) Triton X-100, pH = 7.0), and the nuclei suspension was filtered through a 40 μm nylon mesh. The nuclei were stained with 50 μl of 4′,6-diamidino-2-phenylindole (10 mg/ml) for 5 min. At least 2,000 nuclei were analyzed, and three samples were collected per plant. Leaves sampled from a known diploid plant of *P. tomentosa* (2n = 2x = 38) were taken as a control. The plant DNA *C*-value of *Populus* is 0.46 pg (Kew *C* – value database). The standard peak was set to appear at about channel 50 relative fluorescent intensity. Therefore, when the peaks appeared at channel 75 relative fluorescent intensity, they were considered putative triploids.

We confirmed the ploidy level of each plantlet by somatic chromosome counting. Stem tips were collected and pretreated in a saturated solution of paradichlorobenzene for 2–3 h. Then, the pretreated samples were washed once and fixed in Carnoy’s solution at 4°C for at least 24 h. Subsequently, the fixed stem tips were hydrolyzed in 38% HCl for 25 min at room temperature and then rinsed three times for 10 min each with distilled water. The hydrolyzed samples were stained with Carbol fuchsin, crushed with a coverslip, and observed at 100 × oil lens magnification in an Olympus BX51 microscope (Olympus Inc., Tokyo, Japan).

### Identification of Genetic Constitutions of Colchicine-Induced 2n Pollen

According to the manufacturer’s protocol, DNA was extracted from a 300 mg stored leaf sample of each plant, including the parental poplar lines and their triploid hybrids using the DNeasy Plant Mini Kit (Tiangen Biotech)^1^. The fluorescently labeled TP-M13-SSR method (Schuelke, 2000) was employed in the present study. A forward primer at the 5′ end was attached with a universal primer M13 tail (5′-TGTAAAACGACGGCCAGT-3′) and labeled with four fluorescent substances (6-carboxy-X-rhodamine, 6-carboxy-fluorescein, tetramethyl-6-carboxyrhodamine, or 5-hexachloro-fluorescein). All primers were synthesized by Sangon Inc.^2^ The PCR amplification protocol was as follows: 5 min at 94°C; 10 cycles of 30 s at 94°C, 30 s at the optimal annealing temperature for each SSR marker, and 30 s at 72°C; 25 cycles of 30 s at 94°C, 30 s at 53°C, and 30 s at 72°C; and a final extension of 8 min at 72°C. Then the PCR products were used for capillary electrophoresis fluorescence-based SSR analyses using the ABI 3730xl DNA Analyzer (Applied Biosystems, Foster City, CA, United States), and fragment sizes and peak areas analyzed using GeneMarker software V1.75 (Hulce et al., 2011).

We screened SSR primers from the SSR database^3^ (beginning with “GCPM,” “ORPM”, and “PMGC”) released by the International *Populus* Genome Consortium (IPGC). BLAST analysis in *Populus trichocarpa* v3.0 (Phytozome v12.0)^4^ was done to determinate the location of the SSR loci.

### Statistical Analysis

We analyzed the frequency of colchicine-induced 2n pollen using a univariate general linear model (GLM) to reveal the differences among the meiotic stages, injection times, and the interaction between the meiotic stage and injection time. The data were transformed before analysis of variance and multiple comparisons to account for the heterogeneity of variance. All statistical analyses were performed with SPSS software (version 18.0; SPSS Inc., Chicago, IL, United States).

## Results

### 2n Pollen Production Using 0.5% Colchicine Solution

**FIGURE 1:**
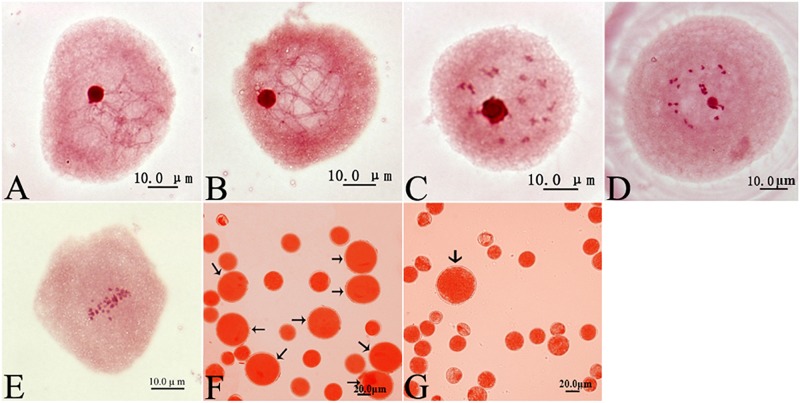
Cytological observations of PMCs, colchicine-induced 2n pollen (arrow) and naturally occurring 2n pollen in *Populus canescens*. **(A)** Leptotene. **(B)** Pachytene. **(C)** Diplotene. **(D)** Diakinesis. **(E)** Metaphase I. **(F)** Colchicine-induced 2n pollen derived from the treatment given 11 injections at pachytene in *P. canescens* (arrow). **(G)** Natural 2n pollen derived from the control group in *P. canescens* (arrow). Scale bar = 10.0 μm **(A–E)** and 20.0 μm **(F,G)**.

After the sampled male buds were injected with 0.5% colchicine solution at leptotene (Figure 1A), pachytene (Figure 1B), diplotene (Figure 1C), diakinesis (Figure 1D), or metaphase I (Figure 1E), the treated male *P. canescens* flower buds were hydroponically cultured in a greenhouse until the catkins matured. As the pollen was released, colchicine-induced 2n pollen was collected from the treated male buds (Figure 1F). A few naturally occurring 2n pollen grains were found in the control group (Figure 1G). The frequency of colchicine-induced 2n pollen ranged from 2.75 to 30.27% (Table 1). We found that the dominant meiotic stage (*F* = 4.801, *P* = 0.009), injection time (*F* = 12.449, *P* = 0.000), and the interaction between the dominant meiotic stage and injection time (*F* = 3.006, *P* = 0.002) had highly significant effects on the frequency of colchicine-induced 2n pollen according to the univariate GLM analysis. Further LSD multiple-comparison tests indicated that the differences in colchicine-induced 2n pollen frequency were significantly higher at pachytene than those at the leptotene, diplotene, or metaphase I stages (*p* < 0.05). The differences in the frequency of colchicine-induced 2n pollen for samples given 11 injections was also significantly higher than that of those given 3, 5, 7, and 9 injections. Hence, the optimal protocol for inducing 2n pollen via colchicine was to give 11 injections of 0.5% colchicine solution at the pachytene stage of the PMCs.

**TABLE 1:** Induction of 2n pollen via a 0.5% colchicine solution in *P. canescens.*

**Dominant meiotic stage of PMCs**	**No. of colchicine injections times**	**Frequency of colchicine-induced 2n pollen (%)**
Leptotene	3	4.79 ± 1.46
	5	6.42 ± 2.13
	7	9.40 ± 1.32
	9	8.19 ± 0.99
	11	10.44 ± 4.41
Pachytene	3	5.46 ± 0.90
	5	6.22 ± 0.89
	7	9.04 ± 2.62
	9	16.70 ± 3.95
	11	30.27 ± 8.69
Diplotene	3	4.18 ± 1.52
	5	5.79 ± 0.60
	7	8.76 ± 0.45
	9	9.48 ± 1.31
	11	9.68 ± 1.76
Diakinesis	3	6.99 ± 1.31
	5	7.81 ± 0.74
	7	8.17 ± 0.56
	9	10.53 ± 0.82
	11	20.36 ± 1.49
Metaphase I	3	2.75 ± 0.43
	5	3.91 ± 1.53
	7	4.34 ± 1.08
	9	11.03 ± 1.23
	11	15.11 ± 4.99
Control		2.08 ± 0.40

### The Effects of Colchicine Treatment on Pollen Morphology

**FIGURE 2:**
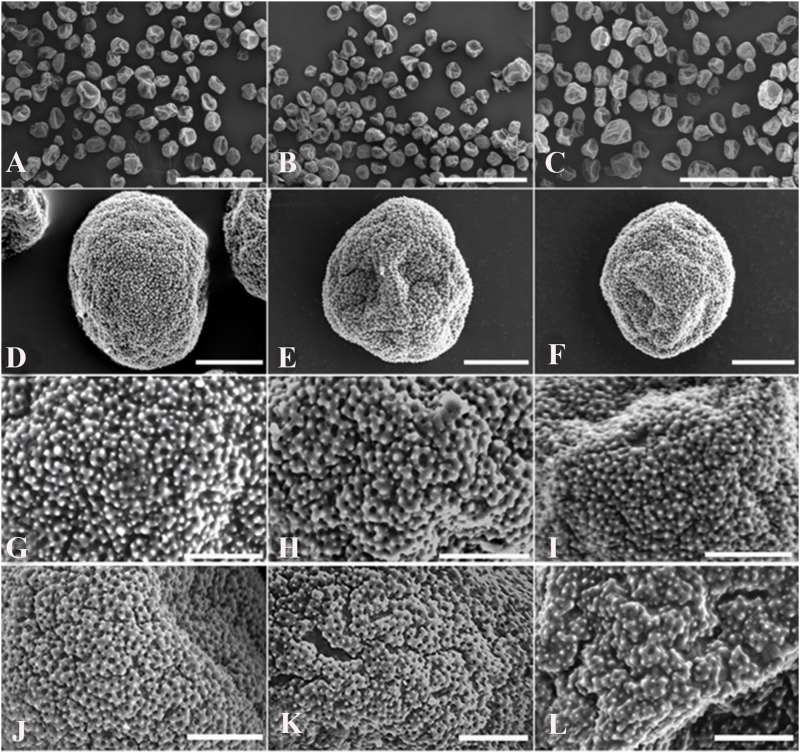
Scanning electron micrographs of *P. canescens* pollen grains derived from untreated and treated male flower buds with 0.5% colchicine at pachytene. **(A)** Morphology of the pollen grains in the control group and natural 2n pollen grains. **(B)** Morphology of the pollen grains derived from the treatment given 5 injections at pachytene and induced 2n pollen grains. **(C)** Morphology of the pollen grains derived from the treatment given 11 injections at pachytene and induced 2n pollen grains. **(D–F)** Ectexine deposition; **(D)** haploid pollen grains in the control group; **(E)** haploid pollen grains derived from the 5 injections treatment at pachytene; **(F)** haploid pollen grains derived from the 11 injections treatment given at pachytene. **(G–L)** Details of the ectexine structure; **(G)** haploid pollen grains in the control group; **(H)** haploid pollen grains derived from the treatment given 5 injections at pachytene; **(I)** haploid pollen grains derived from the treatment given 11 injections at pachytene; **(J)** natural 2n pollen grains; **(K)** 2n pollen grains derived from the treatment given 5 injections at pachytene; **(L)** 2n pollen grains derived from the treatment given 11 injections at pachytene. Scale bar = 100 μm **(A–C)**, 10 μm **(D–F)**, 5 μm **(G–L)**.

The morphology of the pollen was examined via scanning electron microscopy (Figure 2). The pollen grains were non-spherical, with few corrugations and granulated decorations on the surface. 2n pollen was observed in the control group (Figure 2A), the 5 injections group (Figure 2B), and the 11 injections group at pachytene (Figure 2C). The haploid pollen was homogenous, with granulated decorations on the surface, and no aperture was observed in the control or treatment groups (Figures 2D–I). No significant differences were observed in the ectexine structure of haploid pollen between the control and treatment groups because haploid pollen grains were not the outcome of colchicine treatment. However, compared with natural 2n pollen in the control group (Figure 2J), the morphology of the colchicine-induced 2n pollen (Figures 2K,L) showed altered ectexine deposition and some narrow furrows in the ectexine structure.

### Triploid Production by Crossing Colchicine-Induced 2n Pollen

**FIGURE 3:**
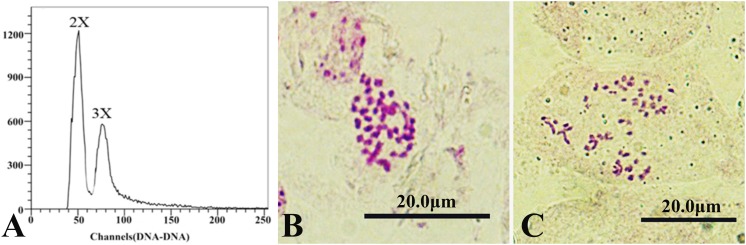
Flow cytometric analysis and somatic chromosome counting of the offspring derived from pollen chromosome doubling induced by colchicine in *P. canescens* (scale bar = 20 μm). **(A)** Flow cytometric analysis of the mixed simple of a diploid plant and a triploid plant; **(B)** Chromosome number of the diploid (2n = 2x = 38); **(C)** Chromosome number of the triploid (2n = 3x = 57).

A total of 6,741 mature seeds were collected from 2 cross-breeding combinations of colchicine-induced 2n pollen and the control group. All seeds were sown and became 4955 young seedlings. Among them, 76 seedlings were derived from the cross between *P. hopeiensis* and *P. canescens*, and 4,879 seedlings were derived from the cross between *P. alba* × *P. glandulosa* and *P. canescens*. The ploidy level of all offspring was examined by flow cytometry, and five putative triploids were detected (Figure 3A). Subsequently, the five putative triploids were confirmed by somatic chromosome counts. The number of chromosomes in the diploid was 38 (2n = 2x = 38, Figure 3B), and the number of chromosomes in the triploid was 57 (2n = 3x = 57, Figure 3C), indicating that the five putative triploids were real triploids. Among the five triploids, two came from *P. hopeiensis* × *P. canescens*, and three were derived from (*P. alba* × *P. glandulosa*) × *P. canescens*.

The number of hybrid offspring and the triploid production rates in the different crosses are shown in Table 2. All triploids were derived from crossing the colchicine-induced 2n pollen with haploid eggs of *P. hopeiensis* or *P. alba* × *P. glandulosa*. No triploid was detected in most of the cross combinations. The highest triploid production rate (16.67%) was observed in the cross between *P. hopeiensis* and *P. canescens*. The average production rate of triploids was 2.35%, which was significantly lower than the frequency of colchicine-induced 2n pollen. These results suggest that the competitive ability of colchicine-induced 2n pollen was significantly weaker than that of haploid pollen.

**TABLE 2:** Triploid production by crossing colchicine-induced *P. canescens* 2n pollen with haploid female gametes in *P. hopeiensis* and *P. alba* × *P. glandulosa.*

**Female parents**	**Frequency of induced 2n pollen (%)**	**Number of seeds**	**Number of seedlings**	**Number of triploids**	**Triploid production rates (%)**
*P. hopeiensis*	30.27	38	6	1	16.67
	20.36	33	9	1	11.11
	16.70	64	21	0	0
	15.11	31	16	0	0
	11.03	52	20	0	0
	10.53	27	4	0	0
*P. alba* × *P. glandulosa*	9.05	912	610	2	0.33
	8.76	1027	864	1	0.12
	6.22	704	563	0	0
	5.79	975	623	0	0
	5.46	1217	964	0	0
	4.34	694	552	0	0
	2.08 (control)	967	703	0	0
	total	6741	4955	5	

### Pollen Germination *in vitro*

**FIGURE 4:**
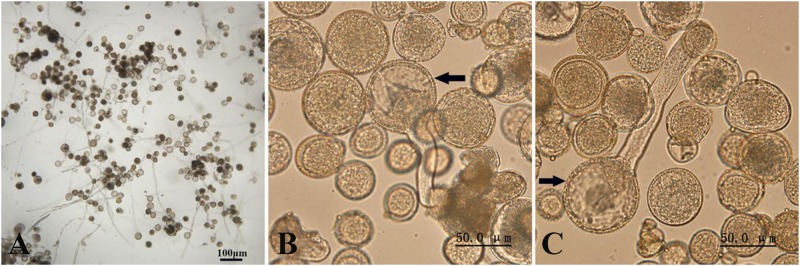
Fresh colchicine-induced pollen of *P. canescens* derived from the treatment given 11 injection times germinated on media. **(A)** Germinated fresh colchicine-induced pollen derived from the treatment given 11 injection times. **(B)** Germinated 2n pollen induced by colchicine (arrow). **(C)** Germinated natural 2n pollen (arrow). Scale bar = 100.0 **(A)** and 50.0 μm **(B,C)**.

**FIGURE 5:**
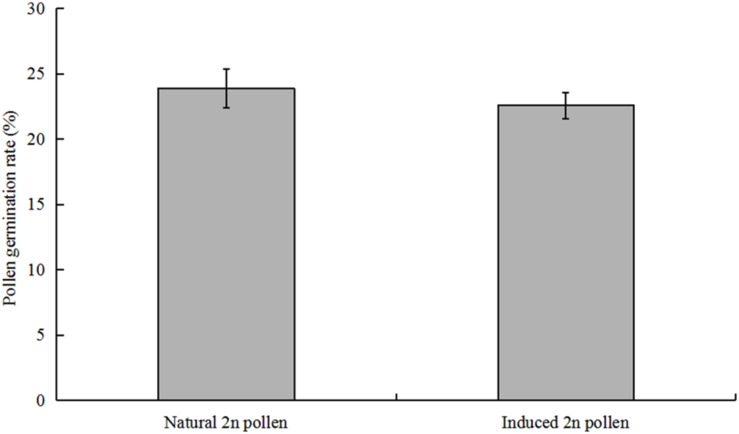
Germination rates of natural 2n pollen derived from the control group and colchicine-induced 2n pollen derived from the treatment given 11 injections with 0.5% colchicine at pachytene in *P. canescens*.

**FIGURE 6:**
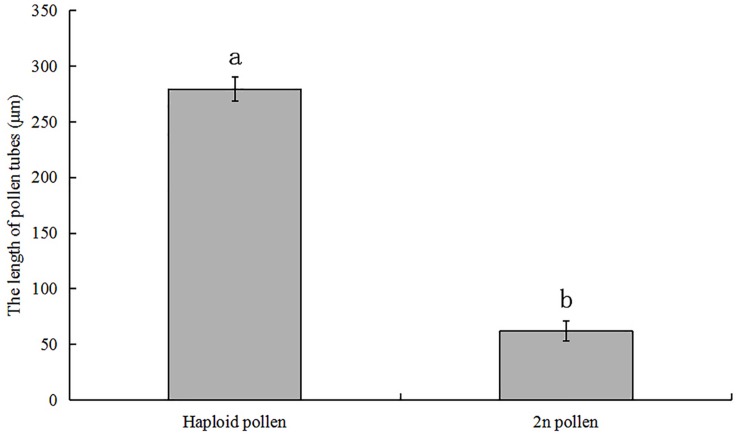
Lengths of the pollen tubes of haploid pollen and colchicine-induced 2n pollen derived from the treatment given 11 injections with 0.5% colchicine at pachytene in *P. canescens*. Lowercase letters represent significant differences at (*P* < 0.05).

The induced pollen of each treatment contained 2n and haploid pollen because the meiosis of PMCs in *P. canescens* is an asynchronous process (Tian M. et al., 2018). We conducted pollen germination experiments *in vitro* to evaluate the effect of colchicine on induced 2n pollen viability. The fresh colchicine-induced pollen grains derived from the 11-injections treatment and the fresh pollen from the control group were used for the germination test on medium containing 50 mg/L calcium chloride and 120 mg/L boric acid. After a 4 h culture, some of the colchicine-induced pollen grains had germinated (Figure 4A). The average pollen germination rate was 27.1%. Some germinated colchicine-induced 2n pollen grains (Figure 4B) and a few germinated natural 2n pollen grains (Figure 4C) were found. The average germination rates of natural 2n pollen and induced 2n pollen were 23.9 and 22.6%, respectively. However, no significant differences were observed in the germination rates between induced 2n pollen and natural 2n pollen (Figure 5). The length of the pollen tube in the induced 2n pollen was 61.9 ± 9.0 μm, which was significantly shorter than that of haploid pollen (279.3 ± 10.9 μm) (Figure 6), suggesting that the slow-growing pollen tube of the colchicine-induced 2n pollen is responsible for the low triploid production rate in *P. canescens*.

### Identification the Genetic Composition of Colchicine-Induced 2n Pollen

**FIGURE 7:**
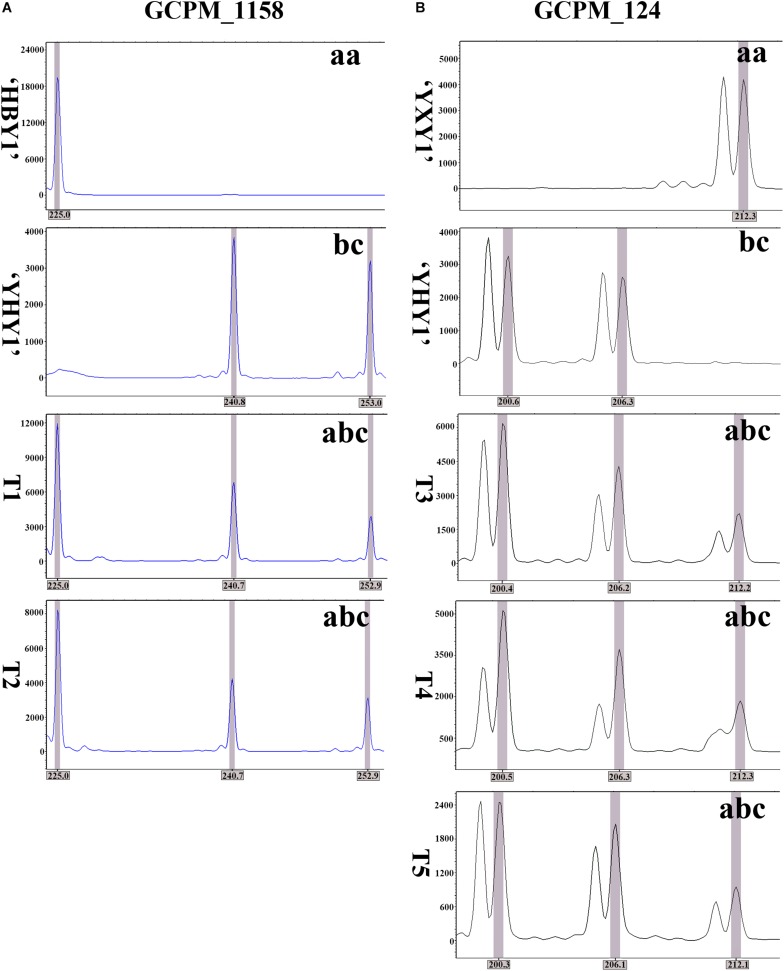
Genetic constitutions of the colchicine-induced 2n pollen in *P. canescens* were revealed by capillary electrophoresis of the loci of primers GCPM_1158 and GCPM_124. **(A)** Allelic configuration in triploid progeny derived from *P. hopeiensis* × *P. canescens* and their parents. T1 and T2 indicate hybrid triploids. **(B)** Allelic configuration in triploid progeny derived from (*P. alba* × *P. glandulosa*) × *P. canescens* and their parents. T3, T4, and T5 also indicate three hybrid triploids.

To identify the genetic composition of colchicine-induced 2n pollen, two pairs of polymorphic SSR primers, GCPM_1158 and GCPM_124, were screened. Table 3 presents the detailed information of the two pairs of SSR primers. The GCPM_124 and the GCPM_1158 loci are located on chromosomes 1 (Chr 01) and 2 (Chr 02), respectively. The capillary electrophoresis results showed that the allelic configuration was, at the GCPM_1158 locus, homozygous in the female parent “HBY1” (225.0 bp, for “aa”), and heterozygous in the male parent “YHY1” (240.8 bp and 253.0 bp, for “b” and “c”) (Figure 7A). The resulting genetic constitution of T1 and T2 triploid hybrids was proven to be “abc.” Therefore, the triploid progeny T1 and T2 could be from FDR-type 2n pollen. Similar to the GCPM_1158 locus, the allelic configuration was, at the GCPM_124 locus, homozygous in female parent “YXY1” (212.3 bp for “aa”), and heterozygous in the male parent “YHY1” (200.6 bp and 206.3 bp, for “b” and “c”) (Figure 7B). The genetic constitution of triploid hybrids T3, T4, and T5, was found to be “abc.” Consequently, triploid progeny T3, T4, and T5 could be inferred to derive from FDR-type 2n pollen.

**TABLE 3:** The detailed information of the two pairs of polymorphic SSR primers and annealing temperature (AT).

**ID of SSR primer**	**Chromosome**	**SSR primer sequence**		**AT(°C)**
		**Forward**	**Reverse**	
GCPM_124	Chr 01	TTTGAGCACTTCAACTACCA	TGTCTTCCCTTAGTCACCAC	53
GCPM_1158	Chr 02	ATGCACTTCCTTCCAAATTA	ATCAGTTCCTTCAGCTTCAA	53

## Discussion

Colchicine is considered an effective chemical mutagen to induce diploid gametes in plants. It inhibits microtubule polymerization by binding to tubulin, and thus it inhibits the mitotic spindle formation resulting in the development of a polyploid cell. Colchicine has been successfully used to induce 2n female gametes by treating embryo sacs, zygotes, and somatic cells, furthermore, a large number of triploids and tetraploids have been generated in *Populus* (Li et al., 2001; Kang et al., 2004; Wang et al., 2010a, b; Cai and Kang, 2011; Xu et al., 2016), *Robinia pseudoacacia* (Ewald et al., 2009), *Eucommia ulmoides* (Zhang et al., 2008), and *Eucalyptus urophylla* (Yang et al., 2018). Colchicine has also been applied to induce 2n pollen production in *Populus* (Li et al., 2014; Zhao et al., 2017, 2019), *Eucommia ulmoides* (Gao et al., 2004), and *Eucalyptus* (Yang et al., 2016). Several triploids have been created by crossing colchicine-induced 2n pollen in *Populus* (Zhao et al., 2017). Therefore, triploid breeding is a promising way to improve *Populus*.

Applying colchicine to PMCs at a suitable stage is vital for inducing 2n pollen (Kang, 2016). The most suitable stage is species dependent. Kang et al. (1999) reported that the most suitable stage for inducing 2n pollen via colchicine varies from leptotene to diplotene in *P. tomentosa* × *P. bolleana*. The leptotene to pachytene stage is the most effective for inducing 2n pollen by colchicine in *P. alba* (Li et al., 2014). In our study, the most effective stage for inducing 2n pollen was pachytene during microsporogenesis of *P. canescens*, which was consistent with the findings reported by Zhao et al. (2019) for *P. deltoid.*

**FIGURE 8:**
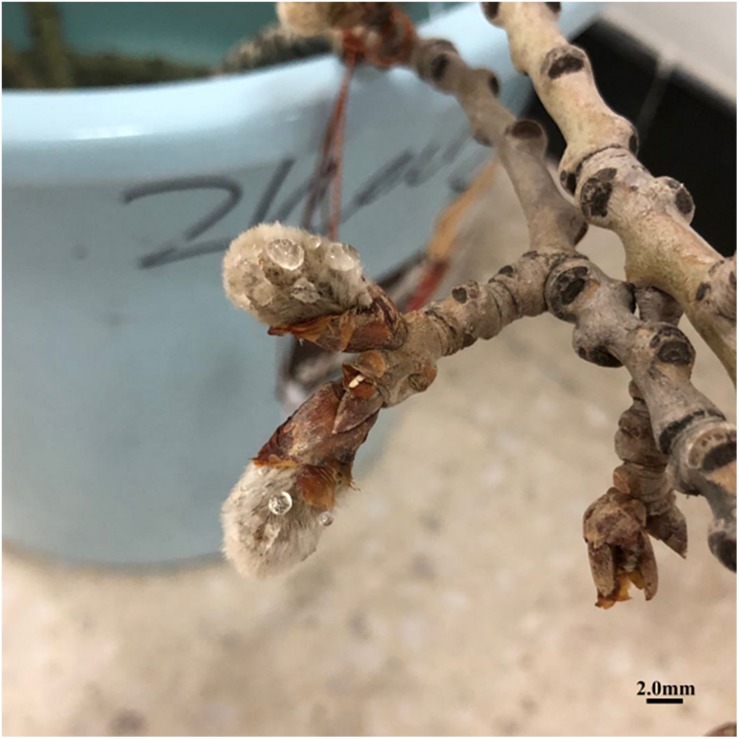
Male flower buds of *P. canescens* after treatment with 0.5% colchicine solution. Scale bar = 2.0 mm.

The number of colchicine injections is key when inducing 2n pollen in *Populus.* According to previous reports, the suitable number of colchicine injections varies from 3 to 5 in *P. tomentosa* × *P. bolleana* for the highest frequency of induced 2n pollen (88%) (Kang et al., 1999). Zhao et al. (2017) documented that 56.5% of induced 2n pollen is achieved when the male *P. tomentosa* buds are given 7 injections of colchicine. In the present study, 11 colchicine injections to the male buds of *P. canescens* at pachytene were used. The highest frequency of induced 2n pollen was 30.27%, which was significantly lower than that of previous studies (Kang et al., 1999; Zhao et al., 2017). At least two aspects are responsible for the lower frequency of induced 2n pollen. The meiotic process of the PMCs in different anthers inside the same flower bud is asynchronous (Tian M. et al., 2018). When the meiotic stage of PMCs in *P. canescens* was at pachytene, the catkin had emerged from the bract scales. The effect of colchicine treatment decreased because the colchicine overflowed and evaporated (Figure 8).

Heterozygosity within a 2n pollen grain depends on the cytological mechanism governing 2n pollen formation. These mechanisms are subdivided into first division restitution (FDR), second division restitution (SDR) (Ramanna and Jacobsen, 2003), and indeterminate meiotic restitution (IMR) (Lim et al., 2001). Pairing and separation of the homologous chromosomes does not occur at meiosis I during FDR, whereas the second division occurs normally with the two sister chromatids of each chromosome moving to opposite poles. Thus, except for cross-over segments, FDR-type 2n pollen retain all homologous parental chromosomes. FDR-type 2n pollen is important when creating heterozygous hybrids due to the highly heterozygous 2n gametes formed (Bretagnolle and Thompson, 1995). In SDR, the pairing and separation of the homologous chromosomes proceed normally during meiosis. In meiosis II, the centromeres of the half-bivalents divide, but the chromatids do not migrate to the poles. Therefore, heterozygosity within SDR-type 2n pollen is lower than that within FDR-type 2n pollen because SDR-type 2n gametes only contain random combinations of sister chromatids. Another restitution mechanism of 2n pollen is IMR. In IMR, unequal distribution of the centromeres of the parental genomes occurs at metaphase I, where some univalents are divided equationally during the first meiotic process, whereas the remaining bivalents are separated before the end of meiosis giving rise to 2n-gametes that cannot be categorized as either FDR or SDR. The IMR mechanism is the process of meiotic restitution to produce fully heterozygous 2n gametes.

Several studies have reported the mechanisms of the formation of 2n gametes in *Populus*. Both FDR-type and SDR-type 2n megaspores are obtained by treating female flower buds with high temperature during megasporogenesis in *P. pseudo-simonii* × *P. nigra* “Zheyin #3” (Wang et al., 2012), *P. adenopoda* (Lu et al., 2013), and *P. tomentosa* (Min et al., 2017). Dong et al. (2015) demonstrated that 74.8% of parental heterozygosity was transmitted by FDR-type 2n female gametes, and 39.6% of parental heterozygosity was transmitted by SDR-type 2n female gametes in *P. pseudo-simonii* × *P. nigra* “Zheyin #3.” However, Lim et al. (2001) and Barba-Gonzalez et al. (2005) reported that IMR-type 2n gametes were detected in interspecific lily hybrids by GISH and FISH. SDR- and PMD -type (post-meiotic genome doubling) 2n gametes were also detected by cytological observation and SSR makers in “Eureka” lemon (Xie et al., 2019). In the present study, the allelic configuration, at the GCPM_1158 and GCPM_124 loci, revealed that the five triploids originated from FDR-type induced 2n pollen. Therefore, the colchicine-induced 2n pollen grains were FDR-type when colchicine treatment occurred before metaphase I.

High-temperature is one of most effective physical mutagenic agents and is widely used to induce 2n gametes in higher plants (Pécrix et al., 2011; Tian M. et al., 2018). Many studies have reported changes in pollen wall structure (Porch and Jahn, 2001; Koti et al., 2005) and a decrease in pollen viability (Porch and Jahn, 2001). For example, 36°C exposure during early meiosis of PMCs leads to a decrease in pollen viability and pollen ectexine defects in induced 2n pollen of *Rosa* (Pécrix et al., 2011). Tian M. et al. (2018) showed a decrease in the pollen production per male flower bud when *P. canescens* buds are exposed to 38°C for 6 h, and the morphology of the induced 2n pollen revealed narrow furrows in the ectexine structure. However, the germination rate of induced 2n pollen was not significantly affected.

It is unknown whether colchicine treatment affects the pollen wall structure and induced 2n pollen viability in *Populus*. Liu et al. (2019) reported that the ectexine structure of *P. tomentosa* induced 2n pollen by giving 3 or 7 colchicine injections at diakinesis of PMCs was similar to that of the natural 2n pollen. In the present study, the morphology of induced 2n pollen was observed by scanning electron microscopy to clarify whether colchicine affected induced 2n pollen viability in *P. canescens*. Some narrow furrows were found in the ectexine structure of colchicine-induced 2n pollen. However, no significant difference in germination rates was detected between colchicine-induced 2n pollen and natural 2n pollen, and five triploids were detected by flow cytometry and somatic chromosome counts, suggesting that colchicine treatment will not result in dysfunction of induced 2n pollen.

In this study, the triploid production rates were significantly lower than the frequency of colchicine-induced 2n pollen, indicating that 2n pollen weakly competed with haploid pollen during fertilization. The *in vitro* pollen germination experiments revealed that the reason the induced 2n pollen was weakly competitive during fertilization was because the pollen tubes of colchicine-induced 2n pollen grew slower than those of haploid pollen. Our findings are consistent with Zhao et al. (2017) in male *P. tomentosa* clone 5088. In a future study, we will focus on revealing the precise reason pollen-tubes of 2n pollen grow so slowly in *P. canescens*.

## Conclusion

The meiotic stage, injection time, and the interaction between the meiotic stage and injection time had highly significant effects on 2n pollen production rates in *P. canescens*. The most effective treatment for inducing 2n pollen was to give 11 injections of 0.5% colchicine solution when PMCs were at the pachytene stage. Colchicine occasionally affected ectexine deposition, and some narrow furrows were detected in the ectexine structure. However, no significant difference in pollen germination rates was observed between natural 2n pollen and colchicine-induced 2n pollen, and five triploids derived from FDR-type induced 2n pollen were generated by crossing colchicine-induced 2n pollen, suggesting that colchicine will not result in dysfunction of colchicine-induced 2n pollen. Slower growth of 2n pollen tubes was responsible for the lower triploid production rate.

## Data Availability Statement

All datasets generated for this study are included in the article/supplementary material.

## Author Contributions

PZ designed the experiments and edited the language of the manuscript. QZ, JW, YS, and ZZ performed the experiments. QZ performed the data analysis and wrote the manuscript. ML contributed the tools for analysis. All authors read and approved the final manuscript.

## Conflict of Interest

The authors declare that the research was conducted in the absence of any commercial or financial relationships that could be construed as a potential conflict of interest.

## Footnotes

1 http://www.tiangen.com/en/?app.html

2 http://www.life-biotech.com/sangonEn.html

3 http://popgenie.org

4 https://phytozome.jgi.doe.gov/pz/portal.html
